# Gut Microbiome Profiles in Colonizations with the Enteric Protozoa *Blastocystis* in Korean Populations

**DOI:** 10.3390/microorganisms10010034

**Published:** 2021-12-24

**Authors:** Moon-Ju Kim, Yu Jeong Lee, Tae-Jong Kim, Eun Jeong Won

**Affiliations:** 1 Department of Parasitology and Tropical Medicine, Chonnam National University Medical School, Hwasun 58128, Korea; 4_chance@naver.com (M.-J.K.); ujeong1202@daum.net (Y.J.L.); 2 Department of Rheumatology, Chonnam National University Medical School and Hospital, Gwangju 61469, Korea; 3 Department of Laboratory Medicine, Chonnam National University Hwasun Hospital, Hwasun 58128, Korea

**Keywords:** *Blastocystis*, Korean, healthy gut microbiota

## Abstract

The influence of unicellular eukaryotic microorganisms on human gut health and disease is largely unexplored. *Blastocystis* species commonly colonize the gut, but their clinical significance and ecological role are unclear. We evaluated the effect of *Blastocystis* colonization on the fecal microbiota of Koreans. In total, 39 *Blastocystis*-positive and -negative fecal samples were analyzed. The fecal microbiome was assessed by targeting the V3–V4 region of the bacterial 16S ribosomal gene. Bacterial diversity was greater in the *Blastocystis*-positive than in the *Blastocystis*-negative group. The bacterial community structure and phylogenetic diversity differed according to the presence of *Blastocystis*. The mean proportions of *Faecalibacterium* species and Ruminococcaceae were larger in the *Blastocystis*-positive group, and that of *Enterococcus* species was larger in the *Blastocystis*-negative group. Linear discriminant analysis showed that *Faecalibacterium, Prevotella 9*, Ruminococcaceae UCG-002, Muribaculaceae, Rikenellaceae, Acidaminococcaceae, *Phascolarctobacterium*, and Ruminococcaceae UCG-005 were highly enriched in the *Blastocystis*-positive group, whereas *Enterococcus hirae, Enterococcus faecalis, Enterococcus durans*, Enterococcaceae, Lactobacillales, and Bacilli were highly abundant in the *Blastocystis*-negative group. Overall, our results enlighten the notion that *Blastocystis* colonization is associated with a healthy gut microbiota.

## 1. Introduction

The mammalian gut contains a microbial community, defined as the microbiome, which includes bacteria, archaea, viruses, phages, yeast, and fungi [1]. The microbiome components have complex roles in gut homeostasis and host health, such as immune system modulation and protection against disease [2,3]. The disruption of the gut ecosystem can lead to gastrointestinal and systemic diseases, such as obesity, diabetes, inflammatory bowel disease, and autoimmune disease [2,3]. Based on this notion, the modulation of gut homeostasis has been recognized as a novel therapeutic target. The plethora of studies documenting the role of the microbiome in human health and disease has directed considerable attention to the factors that influence microbiome diversity and composition [4]. Exploration of the relationship between human-associated gut protists and the resident gut bacterial community has only recently begun.

Advances in DNA-based technologies for the detection and differentiation of intestinal parasites have facilitated exploration of how parasites affect our lives [5]. Luminal intestinal parasitic genera, such as *Blastocystis*, *Dientamoeba*, and *Entamoeba*, are more common in healthy individuals than in patients with functional gastrointestinal disorders [6], indicating that some parasites are beneficial, rather than detrimental, to the host [7]. Of them, *Blastocystis* has been emerging to alter the composition and diversity of the gut bacterial microbiota [8,9,10]. A recent Korean study showed that *Blastocystis* was present in diarrheal and non-diarrheal groups, but that the subtype prevalence differed between the groups, suggesting a role as a modulator of the gut microbiota [11].

The pathogenicity of *Blastocystis* is still unclear and debate over whether *Blastocystis* is a pathogen or mutualist should consider environmental, dietary, and geographic factors. To clarify their implication, clinical studies of diverse populations are required. In this study, we investigated the effect of *Blastocystis* colonization on the gut bacterial microbiome in a Korean population. We identified the taxonomic and functional bacterial features of *Blastocystis* colonization and highlight the role of *Blastocystis* as a common eukaryotic commensal affecting the gut ecosystem.

## 2. Materials and Methods

### 2.1. Collection of Fecal Samples and Blastocystis Screening

Fecal samples were collected from subjects who underwent general checkups or whose gastrointestinal cultures were submitted to the laboratory in accordance with the guidelines, and with the approval, of the Institutional Review Board of Chonnam National University Hospital and Chonnam National University Hwasun Hospital (IRB CNUH-2015-052, CNUHH-2020-045). The samples were transferred immediately to our clinic on ice and stored at −80 °C until processing. Fecal genomic DNA was extracted as described previously using the Cica Geneus^®^ DNA Prep Kit (Kanto Chemical, Tokyo, Japan) according to the manufacturer’s instructions. Firstly, *Blastocystis* screening was conducted by PCR using the Blast-505–532 (5′-GGA GGT AGT GAC AAT AAA TC-3′) and Blast-998–1017 (5′-TGC TTT CGC ACT TGT TCA TC-3′) primers [11], and then amplified PCR products were confirmed by sequencing with the same PCR primers. In addition, *Blastocystis*-negative samples were verified by Real-time PCR using AllplexTM Gastrointestinal Panel-Parasite Assay (GIPPA) (Seegene, Seoul, Korea) [12]. In total, 39 *Blastocystis*-positive (*n* = 23) and *Blastocystis*-negative (*n* = 16) samples were analyzed.

### 2.2. Metagenomic Analysis

PCR amplification was performed using 16S universal primers targeting the V3–V4 region of the bacterial 16S ribosomal gene. The sequencing fragments were detected using MiSeq technology by Macrogen (Seoul, Korea). The targeted metagenomic sequences from the microbiota were analyzed using the bioinformatics pipeline established by Vaiomer (Labège, France) according to the FROGS guidelines. Low-depth samples (with <9000 sequences) were removed from the analysis. The sequences were trimmed, merged, and clustered into operational taxonomic units (OTUs) using CLC Genomics Workbench v.10.1.1 and CLC Microbial Genomics Module v.2.5 (Qiagen, Hilden, Germany). A taxonomic assignment of these sequences was performed based on the NCBI taxonomy database, with an OTU cutoff of 3%. The most abundant sequences were considered to be representative of each cluster and were assigned taxonomy level–based CLC Microbial Genomics default values. To create an OTU table, we first performed multiple sequence alignment with MUSCLE v.3.8.31 with the pre-aligned 16S and 18S data from the SILVA v.132 database. Chimeric sequences were identified and removed, and clustering was performed using the UPARSE protocol. Alpha diversity metrics (richness and Shannon’s index) were calculated using the phyloseq R package based on rarefied OTU counts. The beta diversity index was defined as the difference between the total number of species in the two groups and the number of species common to both groups. The exploratory principal coordinate analysis (PCoA) of beta (between-sample) diversity was performed based on the Bray-Curtis measure of dissimilarity. For the hierarchical cluster analysis, Bray-Curtis metrics and complete linkage clustering were implemented. At deeper taxonomic levels (from the phylum to the genus level), we performed linear discriminant effect-size (LEfSe) analysis based on the non-parametric factorial Kruskal–Wallis rank sum test to detect bacterial taxa with significantly different abundance between responders and non-responders. Next, LEfSe was conducted to estimate the effect size of each differential taxon abundance based on the following criteria: linear discriminant analysis (LDA) value > 4.0; *p* < 0.05. Volcano plots were created to show estimated log2-fold differences in OTU abundance between responders and non-responders.

### 2.3. Statistical Analysis

Alpha diversity metrics were compared by Mann–Whitney tests; for comparisons involving more than two groups, Bonferroni’s correction was applied. The LDA was performed, and the cladogram were generated at http://huttenhower.sph.harvard.edu/galaxy/ (accessd date: 3 December 2021). The two-tailed Student’s *t*-test and *χ*^2^ test were used to assess differences in the phenotypic characteristics of the microbiome using Prism software (GraphPad Software Inc., San Diego, CA, USA).

## 3. Results

### 3.1. Blastocystis Colonization Is Associated with Increased Diversity of Gut Bacterial Microbiota

**Figure 1:**
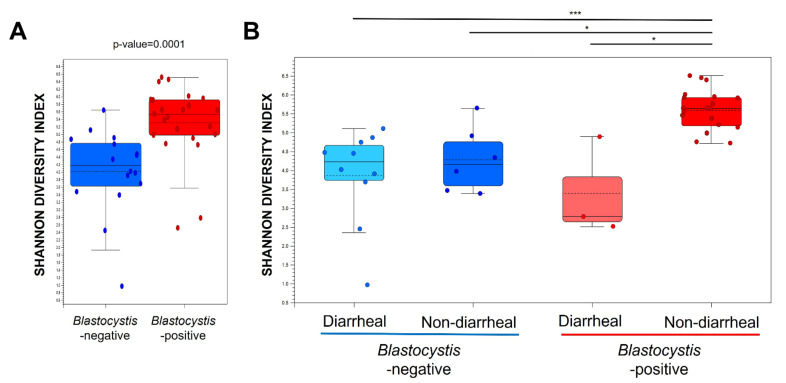
Boxplots of observed OTU richness and Shannon diversity indices for *Blastocystis* colonization and diarrheal type. (**A**) *Blastocystis* (red dots) and the absence thereof (blue dots). (**B**) *Blastocystis*-negative diarrheal (sky-blue dots) and non-diarrheal (blue dots) samples; *Blastocystis*-positive diarrheal (orange dots) and non-diarrheal (red dots) samples. Statistical analysis was performed by a Mann–Whitney U-test. Plotted are interquartile ranges (IQRs; boxes), medians and means (dark lines and dotted lines, respectively, in the boxes), and the lowest and highest values within 1.5-fold of the IQR of the first and third quartiles (whiskers above and below boxes): *, *p* < 0.05, ***, *p* < 0.001.

**Figure 2:**
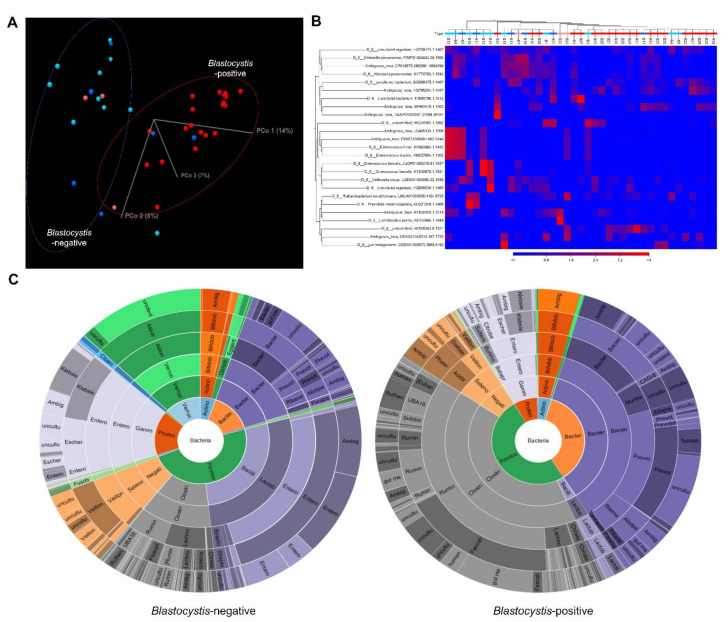
Gut microbiome composition according to *Blastocystis* colonization. (**A**) Unweighted beta diversity analysis showed that the overall bacterial community structure and phylogenetic diversity of the *Blastocystis*-negative (blue and sky-blue) and *Blastocystis*-positive (red and orange) groups were distinct. Blue dots: non-diarrheal *Blastocystis*-negative samples; sky-blue dots: diarrheal *Blastocystis*-negative samples; red dots: non-diarrheal *Blastocystis*-positive samples; orange dots: diarrheal *Blastocystis*-positive samples. (**B**) Heatmap showing operational taxonomic unit abundance in the *Blastocystis*-negative (blue) and *Blastocystis*-positive (red) groups. (**C**) Of note, the Clostridia class and the Ruminococcaceae family were more abundant in the *Blastocystis*-positive group, while the Bacilli class and the Enterobacteriaceae family were noticed in the *Blastocystis*-negative group.

A total of 4,183,214 good-quality reads with a mean length of 301 base pairs was generated. The alpha diversity (Shannon diversity index) is shown in a boxplot in Figure 1. The Shannon diversity index was significantly higher in the *Blastocystis*-positive group than in the *Blastocystis*-negative group; however, it did not significantly differ in accordance with the presence or absence of diarrhea. To explore the differences in the overall microbial community composition across the two main groups of patients, the phylogenetic taxonomic Bray–Curtis dissimilarities were calculated. The unweighted beta diversity analysis also showed that the overall bacterial community structure and phylogenetic diversity differed between the *Blastocystis*-negative and -positive groups (Figure 2A,B). Statistical values obtained by the PERMANOVA test are significant (*p* < 0.001).

### 3.2. Gut Microbiome Composition According to Blastocystis Colonization

**Figure 3:**
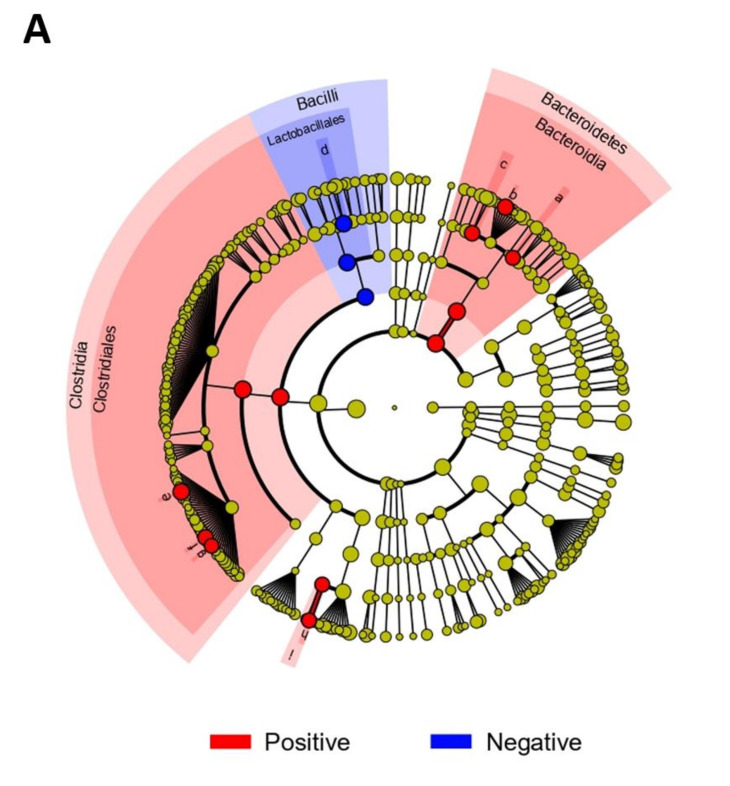

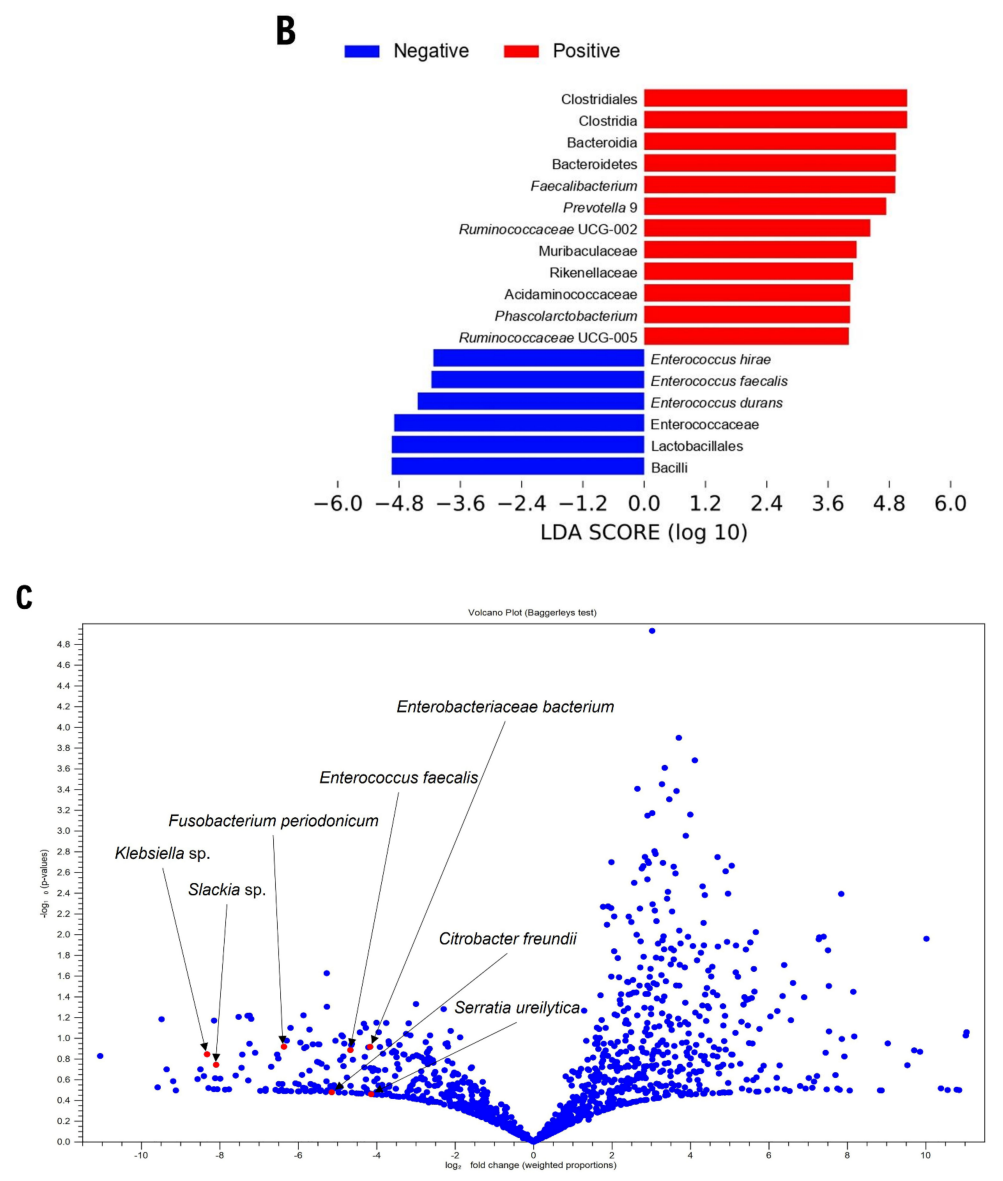
Bacterial taxa associated with the presence or absence of *Blastocystis* according to linear discriminant analysis (LDA) effect size (LEfSe). (**A**) LEfSe taxonomic cladogram showing differences in fecal taxa. Dot size is proportional to taxon abundance. Letters correspond to the following taxa: (a) Muribaculaceae, (b) *Prevotella 9*, (c) Rikenellaceae, (d) Enterococcaceae, (e) *Faecalibacterium*, (f) Ruminococcaceae UCG-002, (g) Ruminococcaceae UCG-005, (h) *Phascolarctobacterium*, and (i) Acidaminococcaceae. (**B**) LDA scores of differentially abundant taxa in the fecal microbiome of the *Blastocystis*-negative (blue) and *Blastocystis*-positive (red) groups. Length denotes effect size. *p* = 0.05, Kruskal–Wallis test; LDA score > 4.0. (**C**) Volcano plot showing bacterial taxa related to *Blastocystis* colonization.

The bacterial composition of fecal samples from *Blastocystis*-positive and *Blastocyst-is*-negative patients was further examined. At the class level, Clostridia were more abundant in the *Blastocystis*-positive group and Bacilli were more abundant in the *Blastocystis*-negative group (Figure 2C and Figure S1). At the family level, Ruminococcaceae were more abundant in the *Blastocystis*-positive group and Enterobacteriaceae were enriched in the *Blastocystis*-negative group. At the genus level, the *Blastocystis*-positive group exhibited a greater abundance of *Faecalibacterium* species, whereas *Bacteroides* and *Enterococcus* species were enriched in the *Blastocystis*-negative group. The mean proportions of *Faecalibacterium* species, *Prevotella 9*, and Ruminococcaceae UCG-002 were larger in the *Blastocystis*-positive group than in the *Blastocystis*-negative group (*Faecalibacterium* species, 19.3% vs. 2.4%, *p* < 0.001; *Prevotella 9*, 13.1% vs. 2.6%, *p* = 0.0065; Ruminococcaceae UCG-002, 6.2% vs. 1.0%, *p* = 0.0007). The *Blastocystis*-negative group had a larger proportion of *Enterococcus* species than did the *Blastocystis*-positive group (17.3% vs. 0.1%, *p* = 0.0114; Figure S2). *Bifidobacterium* species were enriched in the diarrheal, but not the non-diarrheal, *Blastocystis*-positive group. LDA was performed to identify bacterial taxa associated with the *Blastocystis* colonization (Figure 3). Of them, *Faecalibacterium*, *Prevotella 9, Ruminococcaceae* UCG-002, Muribaculaceae, Rikenellaceae, Acidaminococcaceae, *Phascolarctobacterium*, and Ruminococcaceae UCG-005 were highly enriched in the *Blastocystis*-positive group, whereas *Enterococcus hirae, Enterococcus faecalis, Enterococcus durans*, Enterococcaceae, Lactobacillales, and Bacilli were enriched in the *Blastocystis*-negative group. A volcano plot also showed that several bacterial species, such as *Enterobacteriaceae bacterium, Enterococcus faecalis, Fusobacterium periodonicum*, *Citrobacter freundii*, *Serratia ureilytica*, *Klebsiella* sp., and *Slackia* sp., were associated with the *Blastocystis*-negative group.

## 4. Discussion

The diversity and bacterial species composition of the microbiota of healthy individuals differ from those of patients with metabolic or infectious diseases. This study provides insight into the association between *Blastocystis* and gut health in the Korean population. *Blastocystis* was associated with greater microbiota richness and diversity, indicating that *Blastocystis* is a potential biomarker of a healthy gut or promoter of gut microbiome eubiosis [8,13]. The high richness and enterotype distribution in *Blastocystis* carriers indicate that *Blastocystis* is a component of a stable, resilient, and health-associated microbiota; subtype-level analysis revealed important differences within the genus. In agreement with Audebert et al. [8], our cohort showed greater abundance of the class Clostridia and families Ruminococcaceae and Prevotellaceae in *Blastocystis*-positive patients, and of Enterobacteriaceae in *Blastocystis*-negative patients. *Prevotella* species are dominant colonizers of the human gut in non-westernized populations that consume plant-rich diets [14]. *Blastocystis* colonization is thus potentially important in the gut microbiota architecture. Among butyrate-producing bacteria, the relative abundance of *Faecalibacterium*, but not that of *Roseburia*, was high in our cohort. Differences among studies may be, in part, a result of differences in the techniques used to analyze samples, or in geographic, racial/ethnic, or dietary factors. Such inter-study discrepancies reflect the need to evaluate *Blastocystis* in a context-specific manner, accounting for the effects of subtype, inflammation, geographical setting, the dietary factor, and other covariates that influence microbiome composition and diversity [14,15]. In addition, differences in gut bacterial richness were less pronounced in the *Blastocystis*-negative group, regardless of diarrheal status. This finding supports the notion that gut bacterial richness may not be indicative of a healthy gut [16]. The abundance of several *Enterococcus* species, Enterococcaceae, and Bacilli correlated negatively with *Blastocystis* colonization. These results are in accordance with the notion that the relative abundance of Enterobacteriaceae or Enterococcaceae correlates positively with gastrointestinal or systemic inflammation [17]. Our data on the correlation of a heathy microbiome, rich in different species of bacteria, and the presence of *Blastocystis* might be due to gut colonization by this predator protozoa species due to its grazing activity on the bacterial substrate. Together, our results indicate that *Blastocystis* is associated with a healthy gut microbiota.

Fecal microbiota transplantation (FMT) is used to treat several diseases, including recurrent *Clostridium difficile* infection and inflammatory bowel disease [18]. Safe FMT requires careful donor selection via questionnaire administration, interviewing, blood testing, and stool examination [19], but no consensus on the effect of luminal parasites, such as *Blastocystis*, has been reached. Our data partly suggest that colonization may be used as one of the markers for the selection of donors with healthy guts. This notion should be also supported by further studies with a larger population subset.

A recent population-level analysis assessed potential links between *Blastocystis* subtypes and microbiota–host covariates and quantified microbiota differentiation relative to subtype abundance [20]. In that study, the abundance of subtypes ST3 and ST4 was associated inversely with the relative abundance of *Akkermansia*, suggesting that these subtypes are associated with a less-healthy host phenotype. In a previous Korean study, subtype ST3 was the major clone (78.3%); thus, we did not focus on the associations of the gut microbiota with specific subtypes. The relative abundance of *Akkermansia* (>2%) was greater in the *Blastocystis*-negative group (25.0%, 4/16 patients) than in the *Blastocystis*-positive group (4.3%, 1/23 patients). *Akkermansia* is considered to be a health-promoting gastrointestinal isolate and may interact with *Blastocystis* to promote a healthy gut phenotype.

This pilot study was subject to several limitations. First of all, our dataset is too small to be representative of a true population subset and needs further longitudinal studies enrolling larger numbers of subjects with diverse populations. Second, we did not analyze the associations of *Blastocystis* subtypes with the gut microbiome due to the small size of the subgroups. Third, we did not evaluate the role of *Blastocystis* in the structuring of the microbiota or its contribution to intestinal homeostasis. Further longitudinal metagenetic studies in humans or animal models are thus needed. Regardless, our data could serve as the basis for biomarkers of a healthy microbiota and pave the way for an in-depth understanding of the role of *Blastocystis* in the gut. Further insight into the pathophysiological relevance of *Blastocystis* may lead to the development of innovative therapeutic solutions, such as supplementation with probiotics.

## Data Availability

Data sharing not applicable as no datasets generated and/or analyzed for this study. Data are available upon reasonable request. All data relevant to the study are included in the article or uploaded as supplementary information.

## Supplementary Materials

The following are available online at https://www.mdpi.com/article/10.3390/microorganisms10010034/s1, Figure S1. Stepwise distributions of the gut microbiome according to the colonization of Blastocystis or not. Class-Family-Genus-Species level. At the class level, Clostridia were more abundant in the Blastocystis-positive group and Bacilli were more abundant in the Blastocystis-negative group. At the family level, Ruminococcaceae were more abundant in the Blastocystis-positive group and Enterobacteriaceae were enriched in the Blastocystis-negative group. At the genus level, the Blastocystis-positive group exhibited a greater abundance of Faecalibacterium species, whereas Bacteroides and Enterococcus species were enriched in the Blastocystis-negative group. Stacked bars were generated using the operational taxonomic unit files by CLC Genomics Workbench v. 10.1.1 and CLC Microbial Genomics Module v. 2.5 (Qiagen, Hilden, Germany). Figure S2. Potential markers of Blastocystis colonization. At the genus level, the mean proportions of *Faecalibacterium* species, *Prevotella 9*, and Ruminococcaceae UCG-002 were larger in the *Blastocystis*-positive group than in the *Blastocystis*-negative group (*Faecalibacterium* species, 19.3% vs. 2.4%, *p* < 0.001; *Prevotella 9*, 13.1% vs. 2.6%, *p* = 0.0065; Ruminococcaceae UCG-002, 6.2% vs. 1.0%, *p* = 0.0007). The *Blastocystis*-negative group had a larger proportion of *Enterococcus* species than did the *Blastocystis*-positive group (17.3% vs. 0.1%, *p* = 0.0114).
