# Supplementary material for: Gut Microbiome Profiles in Colonizations with the Enteric Protozoa Blastocystis in Korean Populations
Source: Microorganisms. 2021 Dec 24;10(1):34. doi: 10.3390/microorganisms10010034 (PMC8777631; doi:10.3390/microorganisms10010034)
Supplement: Supplementary file 1 [file microorganisms-10-00034-s001.zip › microorganisms-1443107-supplementary.pdf]

microorganisms

MDPI

Supplementary Material

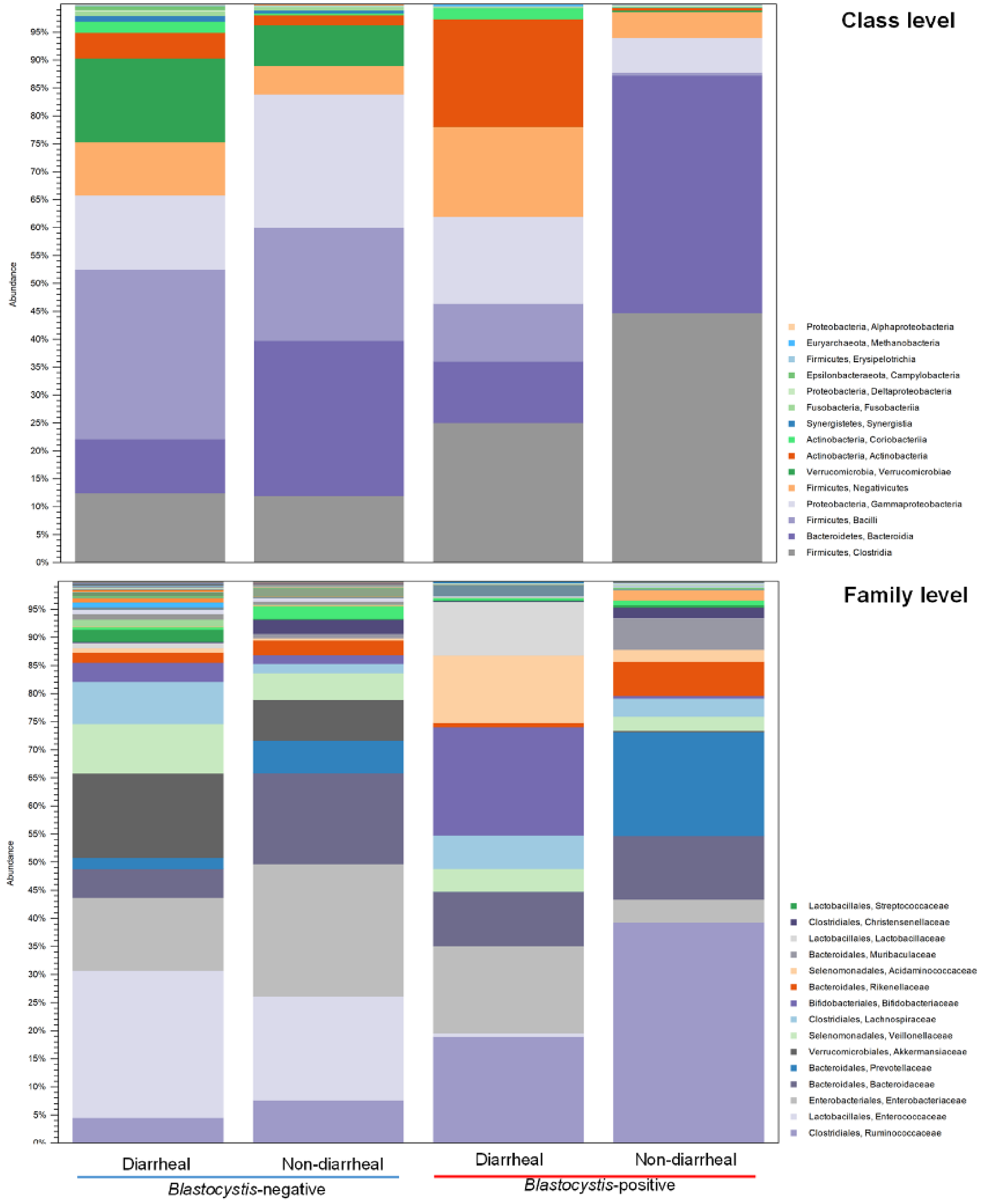

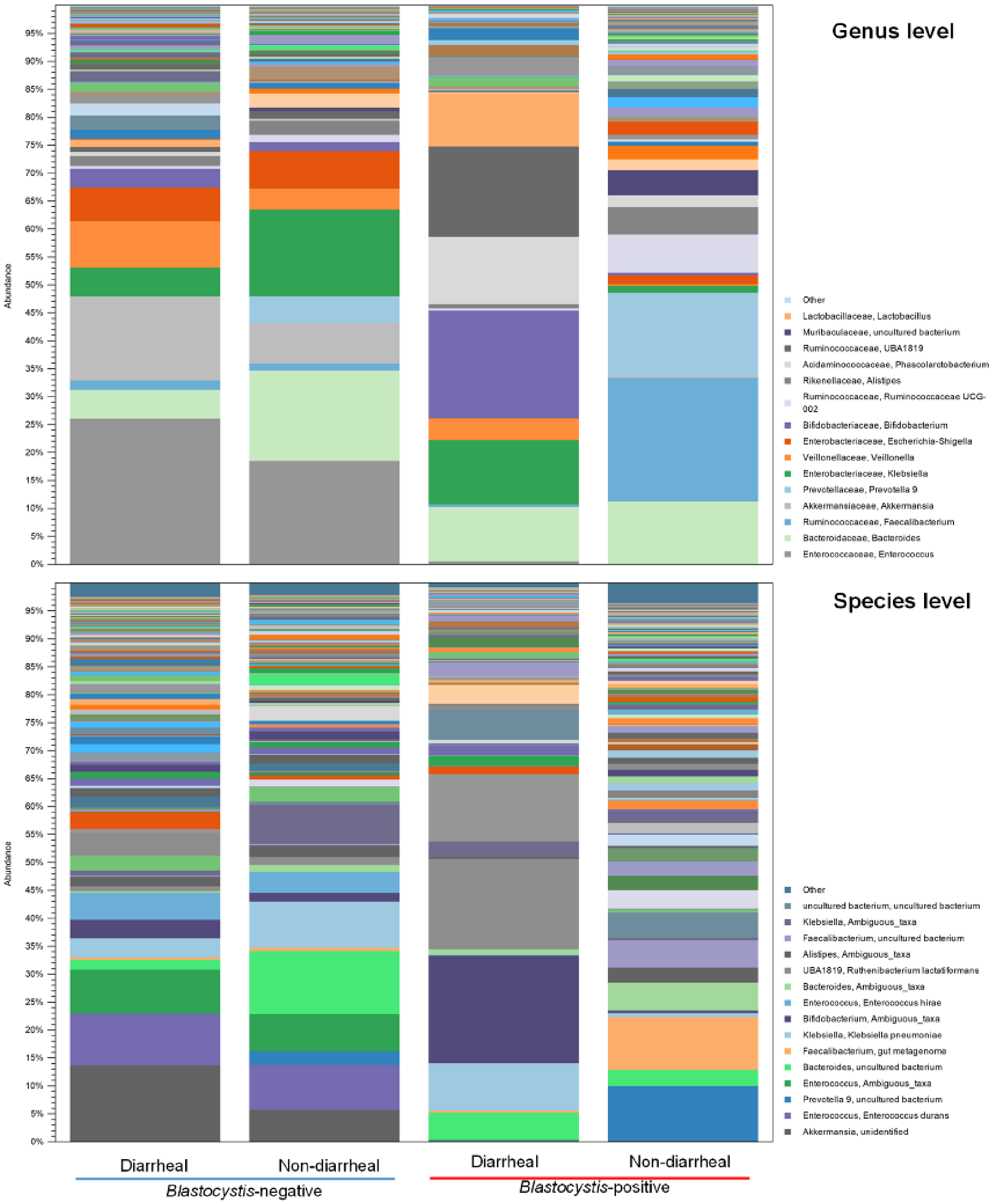

Figure S1. Stepwise distributions of the gut microbiome according to the colonization of *Blastocystis* or not. Class-Family-Genus-Species level. At the class level, Clostridia were more abundant in the *Blastocystis*-positive group and Bacilli were more abundant in the *Blastocystis*-negative group. At the family level, Ruminococcaceae were more abundant in the *Blastocystis*-positive group and Enterobacteriaceae were enriched in the *Blastocystis*-negative group. At the genus level, the *Blastocystis*-positive group exhibited a greater abundance of *Faecalibacterium* species, whereas *Bacteroides* and *Enterococcus* species were enriched in the *Blastocystis*-negative group. Stacked bars were generated using the operational taxonomic unit files by CLC Genomics Workbench v. 10.1.1 and CLC Microbial Genomics Module v. 2.5 (Qiagen, Hilden, Germany).

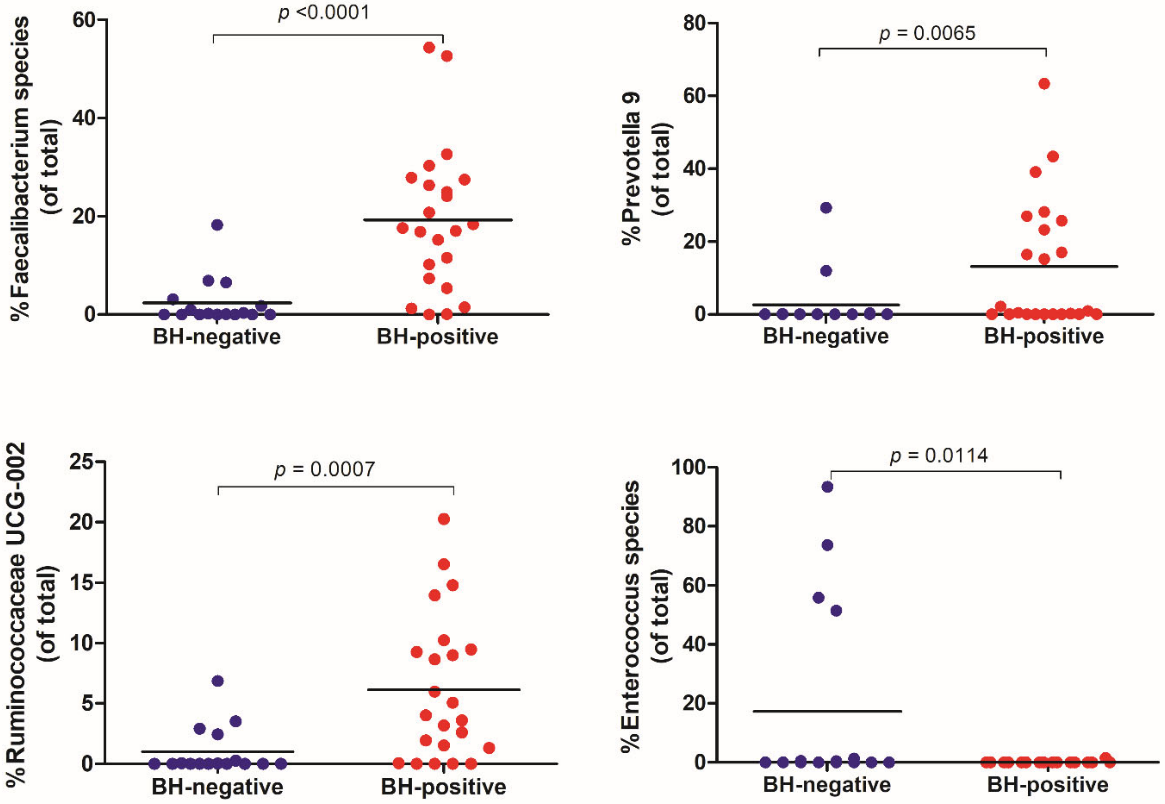

Figure S2. Potential markers of *Blastocystis* colonization. At the genus level, the mean proportions of *Faecalibacterium* species, Prevotella 9, and Ruminococcaceae UCG-002 were larger in the *Blastocystis*-positive group than in the *Blastocystis*-negative group (*Faecalibacterium* species, 19.3% *vs.* 2.4%, $p < 0.001$; Prevotella 9, 13.1% *vs.* 2.6%, $p = 0.0065$; Ruminococcaceae UCG-002, 6.2% *vs.* 1.0%, $p = 0.0007$). The *Blastocystis*-negative group had a larger proportion of *Enterococcus* species than did the *Blastocystis*-positive group (17.3% vs. 0.1%, $p = 0.0114$).
